# The CDO1–ACSM3 Axis Mediates Renal Tubule Lipid Deposition and Injury by Causing Mitochondrial Dysfunction in Lupus Nephritis

**DOI:** 10.3390/cells15050461

**Published:** 2026-03-04

**Authors:** Zibo Zhang, Jinxi Liu, Yunhe Liu, Liwei Wang, Zekun Li, Yan Dong, Yuexin Tian, Xinyan Miao, Qingjuan Liu, Wei Zhang, Huifang Guo, Lingling Xing, Lin Yang, Xiaojuan Feng, Shuxia Liu

**Affiliations:** Key Laboratory of Kidney Diseases of Hebei Province, Department of Pathology, Center of Metabolic Diseases and Cancer Ressearch, Institute of Medical and Health Science, Hebei Medical University, Shijiazhuang 050017, China

**Keywords:** lupus nephritis, CDO1, ACSM3, mitochondrion, lipid deposition

## Abstract

**Highlights:**

**What are the main findings?**

- In Lupus nephritis, CDO1 downregulated ACSM3 expression, leading to mitochondrial morphology disorder and dysfunction, which in turn mediates renal tubular lipid deposition and injury.

**What is the implication of the main finding?**

- CDO1 and ACSM3 are potential therapeutic targets for lupus nephritis.

**Abstract:**

Renal tubular injury plays a critical role in the progression of lupus nephritis (LN); however, the underlying mechanisms remain poorly understood. In this study, we found that CDO1 expression was significantly positively correlated with the degree of renal tubular injury in renal tissues from LN patients. Using in vitro HK-2 and TCMK-1 cells as well as an in vivo MRL/lpr mouse model, we confirmed that knockdown of CDO1 alleviated renal tubular epithelial cell injury and lipid deposition. Mechanistic studies revealed that CDO1 inhibits lipid metabolism by negatively regulating the expression of ACSM3; notably, downregulation of ACSM3 reversed the ameliorative effects of CDO1 knockdown on lipid deposition and cellular injury. Further investigation demonstrated that ACSM3 deficiency mediates lipid deposition by inducing mitochondrial morphological abnormalities and dysfunction. In summary, this study uncovers a novel mechanism by which the CDO1–ACSM3 axis mediates renal tubular lipid deposition and injury in LN through the regulation of mitochondrial function, offering a potential therapeutic target for this disease.

## 1. Introduction

Lupus nephritis (LN) is one of the most common and serious complications of systemic lupus erythematosus (SLE) [1]. Recent clinical observations and basic research have indicated that renal tubular injury is also considered an important indicator for assessing the severity of kidney damage, as well as for predicting treatment response and prognosis [2]. Pathologically, it is mainly characterized by tubular epithelial cell degeneration, necrosis, atrophy, and interstitial fibrosis [1]. Furthermore, the tubular injury could promote renal interstitial fibrosis through activation of profibrotic signaling pathways and, in turn, led to the loss of renal function [3]. In addition, damaged renal tubular epithelial cells can secrete various proinflammatory factors and chemokines that recruit immune cells, thereby amplifying the local inflammatory response and contributing to persistent injury [4]. These findings highlight the important role of renal tubular injury in the progression of LN. However, the underlying mechanisms and potential intervention targets remain not fully understood.

To better understand the specific mechanisms underlying tubular cell injury in LN, renal cortex samples from MRL/lpr mice with evident histopathological features of renal tubular damage were collected for proteomic analysis. This approach led to the identification of cysteine dioxygenase type 1 (CDO1) as a significantly upregulated protein. CDO1 is a non-heme Fe(II)-dependent dioxygenase that belongs to the cupin superfamily. In mammals, its expression is predominantly observed in tissues such as adipose tissue, liver, brain, small intestine, and lungs [5,6]. Recent studies have shown that CDO1 expression downregulated in tumor cells, suppressed tumor cell apoptosis, and promoted cell invasion and migration by enhancing the antioxidant capacity of tumor cells [7,8]. In addition, CDO1 was downregulated in non-alcoholic fatty liver disease (NAFLD), and it exacerbated hepatic steatosis by suppressing fatty acid oxidation and mitochondrial function in hepatocytes [9]. Notably, despite its important role in metabolic regulation and oxidative stress, the expression and biological function of CDO1 in kidney diseases, particularly in LN-associated renal tubular injury, remain completely unknown to date.

Typical pathological features of renal tubular injury in LN include apoptosis and lipid deposition. Based on the significant upregulation of CDO1 revealed by proteomic analysis, we posed the following scientific questions: Does CDO1 participate in the regulation of renal tubular injury, especially lipid deposition, in LN, and what is the underlying mechanism? Therefore, this study is the first to investigate the role of CDO1 in renal tubular injury in LN and to elucidate its downstream molecular mechanisms. Our findings demonstrate that increased CDO1 expression induces renal tubular injury in LN, particularly lipid deposition; mechanistically, CDO1 regulates mitochondrial morphology and function by modulating ACSM3 expression, thereby mediating lipid accumulation. Targeted downregulation of CDO1 significantly ameliorated lipid deposition and improved renal function in MRL/lpr mice. These findings provide a new theoretical basis for elucidating the mechanisms of renal tubular injury in LN and offer novel insights for the development of precise therapeutic strategies targeting the renal tubules in this disease.

## 2. Materials and Methods

### 2.1. Patients and Tissue Samples

This study enrolled 30 patients, aged 25 to 50 years, who were diagnosed with class III, IV, or V lupus nephritis (according to the ISN/RPS 2003 classification) at the Department of Nephrology, Second Hospital of Hebei Medical University, between 2020 and 2023. Renal tissue samples were collected from all participants for analysis. At the same time, 10 control kidney tissue samples were obtained from nephrectomy specimens of patients with renal angiomyolipoma or renal cancer. These samples were pathologically confirmed as normal kidney tissue, with no evidence of primary glomerulonephritis, hypertension, diabetic nephropathy, or a history of autoimmune diseases, and were matched for age and sex with the LN group. The above renal tissue were fixed with 4% formaldehyde for immunohistochemistry (IHC) and immunofluorescence (IF) staining.

The plasma samples were collected from five active LN patients who had no immunosuppressive therapy, infections, or other complications and underwent therapeutic plasma exchange. All patients met the 2012 revised criteria for SLE and LN and were diagnosed at Department of Rheumatology of the Second Hospital of Hebei Medical University from 2020 to 2023. Additionally, the equal number of plasma samples were obtained from five age- and sex-matched healthy donors, serving as the control group. This study was approved by the Clinical Research Ethics Committee of the Second Hospital of Hebei Medical University (IRB ID:2023-030).

### 2.2. Proteomic Analysis

Proteomic analysis was performed on the renal cortex of three 28-week-old female MRL/lpr mice and three female MRL/MPJ mice. The criteria for screening differentially expressed proteins were set as a fold change (FC) greater than 1.2 (upregulated by more than 1.2-fold or downregulated to less than 0.83-fold) and a *p*-value less than 0.05. A volcano plot was used to visualize the differential expression of proteins: significantly downregulated proteins were marked in green (FC < 0.83 and *p* < 0.05), significantly upregulated proteins were marked in red (FC > 1.2 and *p* < 0.05), and proteins with no significant differences were marked in blue. The proteomic analysis was conducted by Shanghai Applied Protein Technology.

### 2.3. Human Genome Microarray Analysis

Transcriptomics data were obtained from healthy control kidney biopsy tissues (*n* = 29) and LN renal biopsy tissues (*n* = 64) from a previous study (GSE32591). The expression level of CDO1 and its correlation with tubular injury were analyzed using R software (R version 4.2.1).

### 2.4. Immunohistochemical Staining (IHC)

Immunohistochemistry (IHC) staining was performed as described in our previous study [10]. The following primary antibodies were used: anti-CDO1 (1:200, Proteintech, 12589-1-AP, Rosemont, IL, USA), anti-ACSM3 (1:200, Proteintech, 10168-2-AP), anti-NGAL (1:200, Proteintech, 26991-1-AP), anti-Kim-1 (1:100, Novus, NBP1-76701, Centennial, CO, USA), and anti-ADRP (1:200, Proteintech, 15294-1-AP). Images were captured and quantitatively analyzed following the methods described in [11].

### 2.5. Immunofluorescence (IF)

After antigen retrieval was performed using a pressure cooker, endogenous peroxidase activity was blocked with 3% H_2_O_2_ for 30 min at room temperature. The sections were then blocked with 10% goat serum and incubated with primary antibodies against CDO1 (1:200, Proteintech, 12589-1-AP), AQP1 (1:200, Proteintech, 66805-1-Ig), and COX IV (1:1000, Abcam, ab33985, Cambridge, MA, USA). Following incubation with TRITC-conjugated goat anti-rabbit IgG (H + L) (1:200, Proteintech, SA00007-2), FITC-conjugated goat anti-mouse IgG (H + L) (1:200, Proteintech, SA00003-1), and DAPI (SouthernBiotech, Birmingham, AL, USA), fluorescence images were captured using a laser scanning confocal microscope (Leica, Wetzlar, Germany).

### 2.6. Animals and Groups

Twenty-six-week-old (weighing 25–30 g) female MRL/lpr lupus-prone mice and age- and sex-matched MRL/MPJ mice were obtained from Huachuang Xinnuo Biotechnology (Taizhou City, Jiangsu Province, China). The animals were housed under standard conditions with a regular light/dark cycle and had free access to food and water. To investigate the role of CDO1, mice in the MRL/lpr group were divided and administered via tail vein injection with either 100 μL of adeno-associated virus (AAV) carrying the KSP promoter (1 × 10^12^ pfu/mL; HanBio Biotechnology, Shanghai, China) or an equal volume of normal saline as a control. After 4 weeks, the mice were euthanized, and samples of urine, blood, and renal cortex tissue were collected for subsequent analysis. The specific experimental groupings are detailed in Table S1. All animal experiments were approved by the Animal Care and Use Committee of Hebei Medical University (Approval No. HebMU clars2022163).

### 2.7. Real-Time PCR

Total RNA was extracted from mouse renal cortex tissue using TRIzol reagent (UElandy, U7431, Suzhou, China) following the manufacturer’s instructions. The RNA was then reversed to cDNA using a reverse transcription kit (Yeasen Biotechnology, R323-01, Shanghai, China). Real-time PCR was performed using SYBR Green PCR Master Mix (Yeasen Biotechnology) on a StepOnePlus Real-Time PCR System (Applied Biosystems, Foster City, CA, USA). The specific primers used were as following: CDO1: Forward 5′-CGGACTCCCACTGCTTTCTGAAG-3′ and Reverse 5′-GCTGACGTTCTCTACTCGGTGTAAG-3′; ACSM3: Forward 5′-TTGATAGCCTCCGATGTGATGTGG-3′ and Reverse 5′-GTTGGTGCAGAACAGAAGACAGTG-3′; 18S: Forward 5′-ACACGGACAGGATTGACAGA-3′ and Reverse 5′-GGACATCTAAGGGCATCACAG-3′. The 2^−ΔΔCT^ method was used to normalize the qPCR cDNAs. All experiments were repeated at least in triplicate.

### 2.8. Hematoxylin-Eosin (HE) and Masson Staining

HE and Masson staining were performed as described in our previous article [12]. Five random fields of view were selected from each section, and images were examined using an Olympus microscope for subsequent analysis. Then, the injury of the kidney was quantified and assessed in a blinded manner by two experienced pathologists, including loss of brush border, vacuolation, tubular epithelial cell detachment, tubular dilation, and tubular degeneration. The degree of injury for each indicator was scored on a scale of 0–3: none (0), mild (1), moderate (2), and severe (3) [13].

### 2.9. Western Blotting

Western blot was carried out as described in our previous study [14]. The antibody concentration was anti-CDO1 (1:1000, Abcam, ab232699), anti-Bcl-2 (1:1500, Proteintech, 33799-1), anti-BAX (1:5000, Proteintech, 60627-1), anti-NGAL (1:1000, Abcam, ab125075), anti-Kim-1 (1:1000, NOVUS, NBP1-76701), anti-FN (1:3000, Abcam, ab2413), anti-α-SMA (1:5000, Abcam, ab5694), anti-E-cadherin (1:1000, Abcam, ab231303), anti-Collagen I (1:5000, Proteintech, 67288-1), anti-ACSM3 (1:500, Santa Cruz, sc-377173, Dallas, TX, USA), anti-ADRP (1:1000, Proteintech, 15294-1-AP), anti-GAPDH (1:10,000, Proteintech, 10494-1), anti-Tubulin (1:5000, Proteintech, 14555-1), HRP-conjugated Affinipure Goat Anti-Rabbit IgG(H + L) (1:5000, Proteintech, SA00001-2), HRP-conjugated Affinipure Goat Anti-Mouse IgG(H + L) (1:5000, Proteintech, SA00001-1). The membranes were visualized using the LI-COR Odyssey infrared imaging system (LI-COR Biosciences, Lincoln, NE, USA). All experiments were performed in triplicate.

### 2.10. Biochemical Index Detection

Serum creatinine (scr), blood urea nitrogen (BUN), and 24 h proteinuria levels were measured using commercial kits (C011-2-1, C013-2-1, and C035-2-1) purchased from Nanjing Jiancheng Bioengineering Institute (Nanjing, China) following the manufacturer’s instructions.

### 2.11. Enzyme-Linked Immunosorbent Assay (ELISA)

Human Kim-1 and NGAL in the urine of mice were detected using commercial ELISA kits (ZC-38656 and ZC-39020) obtained from ZCIBIO (Shanghai, China) according to the manufacturer’s protocols.

### 2.12. Oil Red O Staining

The lipid droplet levels were measured using the Oil Red O staining kit (Report, RK1006, Shijiazhuang, China) according to the instruction procedure. The images were captured using an Olympus microscope (OLYMPUS, BX71, Tokyo, Japan).

### 2.13. Cell Culture and Groups

HK-2 cells were purchased from the Chinese Academy of Sciences, Shanghai Institute for Biological Sciences Cell Resource Center (Shanghai, China). TCMK-1 cells were purchased from the iCell Bioscience (Shanghai, China). Both cell lines were cultured as previously described [15]. Following stimulation with plasma from LN patients for 48 h, renal tubular epithelial cells were collected for subsequent experiments. All cell experiments were performed with three biological replicates.

### 2.14. Small Interfering RNA (siRNA) and Plasmid Transfection

siRNA targeting human or mouse CDO1, ACSM3, and the plasmid encoding ACSM3 were obtained from GenePharma Biotechnology. Transfection was performed using GP-transfect-Mate (GenePharma, G04009, Suzhou, China) in Opti-MEM (Gibco, 31985062, Waltham, MA, USA) according to the manufacturer’s instructions. Six hours later, the fresh culture medium was replaced, and subsequent experiments were performed after twenty-four hours.

### 2.15. BODIPY Staining

HK-2 and TCMK-1 cells from different treatment groups were washed with PBS and then incubated with 10 μM BODIPY 493/503 (MedChemExpress, 216434-81-0, New Jersey, NJ, USA) at 37 °C for 15 min. After incubation, the cells were fixed with 4% paraformaldehyde for 20 min and stained with DAPI. Fluorescence images were captured using a laser scanning confocal microscope (Leica, Wetzlar, Germany), and the fluorescence intensity of the lipid droplets was measured with flow cytometry.

### 2.16. Mito-Tracker Green Assay

To observe the morphology of the mitochondria, the HK-2 cells were stained with Mito-Tracker Green (Beyotime, C1048, Shanghai, China) and Hoechst 33342 (Beyotime, C1027) at 37 °C for 30 min. Then, the images were captured using confocal laser scanning microscopy (Leica, Wetzlar, Germany).

### 2.17. Mitochondrial Transmembrane Potential

To observe the mitochondrial transmembrane potential, HK-2 cells were placed in an environment of 37 °C and stained with JC-1 kit (Beyotime, C2006, Shanghai, China) for 30 min. Subsequently, imaging was carried out using a confocal laser scanning microscope from Leica AG (Wetzlar, Germany).

### 2.18. ATP Content Measurement

The ATP content of cultured cells was measured using an ATP assay kit (Nanjing Jiancheng Bioengineering Institute, A095-1-1, Nanjing, China) according to the manufacturer’s protocol.

### 2.19. RNA-Sequencing Analysis

Transcriptome sequencing was supported by OEBiotech (Shanghai, China). Key steps included total RNA extraction, qualification, quantification, library construction, clustering, sequencing, and data analysis. Differentially expressed genes (DEGs) were identified using the DESeq2 R package (R version 4.2.1), with adjusted *p* < 0.05 considered statistically significant. To further explore the functions of DEGs, gene ontology (GO) enrichment analysis was conducted, describing their biological processes, cellular components, and molecular functions based on GO annotations.

### 2.20. Statistical Analysis

Data were expressed as mean ± standard deviation (SD). One-way analysis of variance (ANOVA) and Student’s *t*-test were employed to evaluate statistical significance between groups. Correlation analysis was conducted using Pearson correlation analysis. A *p*-value < 0.05 was considered statistically significant. SPSS 23.0 statistical software (IBM, New York, NY, USA) was used for all the statistical analyses.

## 3. Results

### 3.1. Elevated CDO1 Expression in Renal Tubules of LN Was Significantly Associated with Tubular Injury

**Figure 1:**
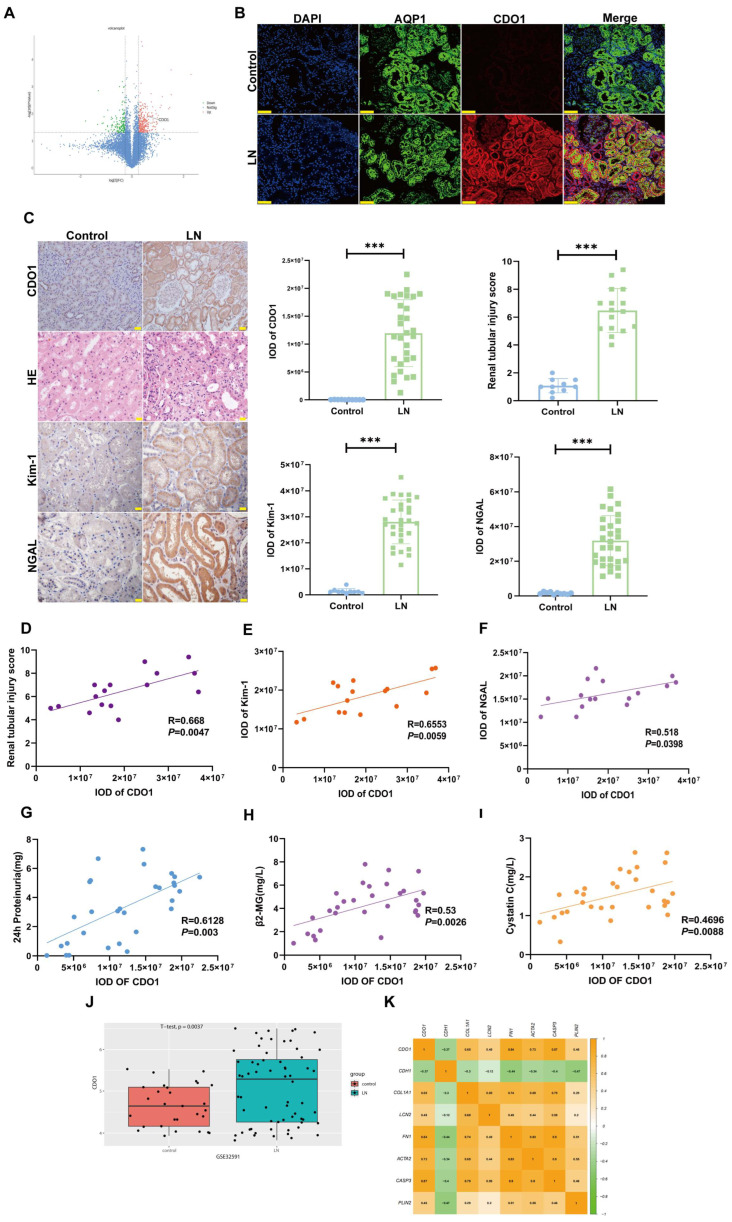
**CDO1 expression was elevated in renal proximal tubules of patients with lupus nephritis and positively associated with tubular injury.** (**A**) Differentially expressed genes in renal tissues between MRL/MPJ and MRL/lpr mice groups (*n* = 3). (**B**) IF showed the co-localization of CDO1 (red) and AQP1 (green) in renal tissues from controls and LN patients. The nucleus is stained with DAPI. Scale bar: 75 μm. (**C**) IHC showed the expression of CDO1, Kim-1, and NGAL in renal tissues of the control group (*n* = 10) and LN patients (*n* = 30) (brown areas indicate positivity). HE staining revealed renal tissue injury in both the control group (*n* = 10) and LN patients (*n* = 16). The IOD value was used for semi-quantitative analysis of IHC results, and the renal tubular injury score was employed for semi-quantitative analysis of HE results. Scale bars: 20 μm and 50 μm. (**D**–**F**) CDO1 expression in renal tubules showed positive correlations with the renal tubular injury score, Kim-1 and NGAL expression of LN patients (*n* = 16). (**G**–**I**) CDO1 expression in renal tubules showed positive correlations with 24 h proteinuria, β2-MG and Cystatin C expression in LN patients (*n* = 30). (**J**) CDO1 mRNA levels in renal tissues between the control group (*n* = 29) and LN patients (*n* = 64) from the GEO database. (**K**) CDO1 expression in renal tissues of the GEO database (control group *n* = 29, LN patients *n* = 64) was positively correlated with renal tubular injury. Data are presented as mean ± SD. *** p < 0.001; (Control vs. LN).

As shown in Figure 1A, proteomic analysis revealed significant changes in the expression of 478 proteins, among which CDO1 was one of the most notably upregulated. Subsequently, we found that the expression of CDO1 increased in the cytoplasm of the renal tubular epithelial cells especially in the proximal tubule of the LN group compared to the control group, following with upregulation of the level of renal tubular injury (Figure 1B,C). Further analysis revealed that CDO1 expression levels in the renal tubules of LN patients were positively correlated with the degree of tubular injury, as well as with urinary levels of β2-microglobulin (β2-MG), cystatin C, and 24 h proteinuria (Figure 1D–I). Transcriptomic datasets from public databases showed that CDO1 mRNA levels were significantly elevated in the kidneys of LN patients compared to healthy controls (Figure 1J). Meanwhile, CDO1 mRNA expression was significantly correlated with markers of tubular injury, epithelial-mesenchymal transition (EMT), apoptosis, and lipid deposition in LN renal tissues (Figure 1K).

### 3.2. Specific Knockdown of CDO1 Expression in Renal Tubular Epithelial Cells Alleviated Renal Tubular Injury In Vivo and In Vitro

**Figure 2:**
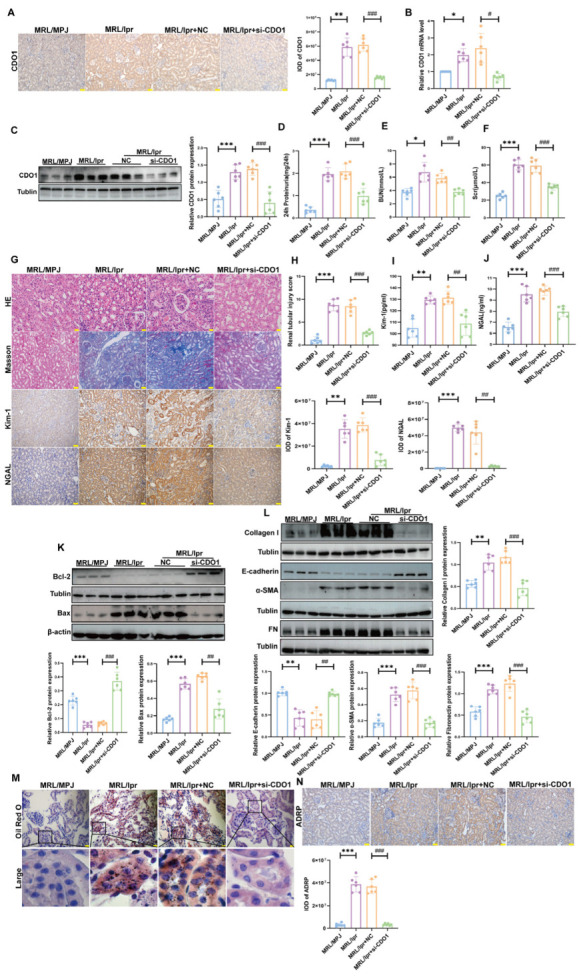
**Specific knockdown of CDO1 expression in renal tubular alleviated renal tubular injury in MRL/lpr mice.** (**A**) IHC showed the expression and localization of CDO1 in MRL/lpr mice (*n* = 6) (brown areas indicate positivity, nucleus is blue). Scale bar: 50 μm. (**B**,**C**) CDO1 knockdown reduced mRNA levels (*n* = 6) and protein expression (*n* = 6) of CDO1 in MRL/lpr mice. (**D**–**F**) CDO1 knockdown decreased 24 h proteinuria, BUN, and Scr levels in MRL/lpr mice (*n* = 6). (**G**) IHC showed the expression of Kim-1 and NGAL in mouse renal tissue (brown areas indicate positivity). HE staining revealed renal tissue injury in mice. Masson staining showed collagen deposition in mouse renal tissue (blue indicates collagen deposition) (*n* = 6). Scale bars: 20 μm and 50 μm. (**H**) Quantitative analysis of renal tubular injury score in MRL/lpr mice after CDO1 knockdown (*n* = 6). (**I**,**J**) Urinary Kim-1 and NGAL levels in MRL/lpr mice decreased after CDO1 knockdown (*n* = 6). (**K**) CDO1 knockdown reduced apoptosis levels in MRL/lpr mice (*n* = 6). (**L**) CDO1 knockdown reduced EMT levels in MRL/lpr mice (*n* = 6). (**M**) CDO1 knockdown alleviated lipid deposition in MRL/lpr mice. The black box indicates the magnified area. The red dot represents the lipid droplet. Scale bar: 20 μm. (**N**) IHC staining showed ADRP expression in MRL/lpr mice (*n* = 6) (brown areas indicate positivity, nucleus is blue). Scale bar: 50 μm. Data are presented as mean ± SD. * *p* < 0.05, ** *p* < 0.01, *** *p* < 0.001 (MRL/MPJ vs. MRL/lpr). ^#^ *p* < 0.05, ^##^ *p* < 0.01, ^###^ *p* < 0.001 (MRL/lpr + NC vs. MRL/lpr + si-CDO1).

To further investigate the role of CDO1 in renal tubular cell injury in LN, we performed in vivo experiments using MRL/lpr mice. First, both mRNA and protein levels of CDO1 were significantly increased in the renal cortex of MRL/lpr mice compared with MRL/MPJ control mice (Figure 2A–C). In addition, the EMT and injury indicators including the expression of Kim-1, NGAL, Bax/Bcl-2, and E-cadherin was significantly changed (Figure 2G–L). Notably, specific knockdown of CDO1 via intravenous injection of Ksp-si-CDO1-AAV markedly reduced 24 h proteinuria, BUN, and Scr levels (Figure 2D–F). Furthermore, the renal tubular injury and interstitial fibrosis were significantly improved compared with MRL/lpr mice (Figure 2G–L). Most importantly, lipid deposition and the expression of ADRP (a lipid droplet marker) were significantly reduced in the renal tubules of CDO1-knockdown mice relative to MRL/lpr mice (Figure 2M,N). Together, these findings demonstrate that knockdown of CDO1 alleviates renal tubular injury and improves kidney function in MRL/lpr mice.

**Figure 3:**
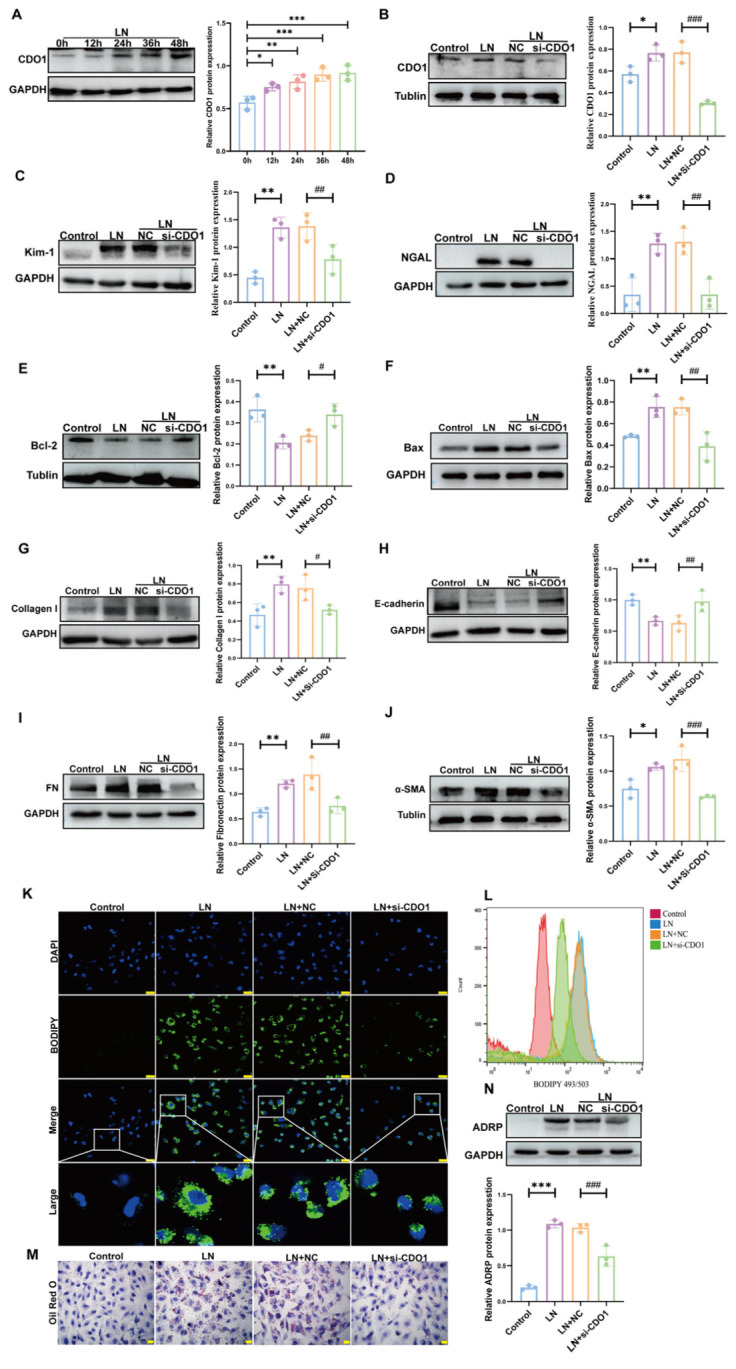
**CDO1 Knockdown alleviated HK-2 cell injury induced by LN plasma.** (**A**) CDO1 expression was elevated in HK-2 cells stimulated with 10% LN plasma (*n* = 3). (**B**) Under the stimulation of 10% LN plasma, siRNA significantly downregulated CDO1 expression (*n* = 3). (**C**,**D**) Knockdown of CDO1 reduced Kim-1 and NGAL protein expression (*n* = 3). (**E**,**F**) Apoptosis levels decreased after CDO1 knockdown (*n* = 3). (**G**–**J**) EMT levels were reduced after CDO1 knockdown (*n* = 3). (**K**) BODIPY staining showed alleviated lipid deposition in cells after CDO1 knockdown. The white box indicates the magnified area. The green dots represent lipid droplets. The nucleus is blue. Scale bar: 25 μm. (**L**) Fluorescence intensity of lipid droplets decreased post CDO1 knockdown. (**M**) Oil red O staining demonstrated reduced lipid deposition in cells after CDO1 knockdown. The red dots represent lipid droplets. The nucleus is blue. Scale bar: 20 μm. (**N**) Knockdown of CDO1 reduced ADRP protein expression (*n* = 3). Data are presented as mean ± SD. * *p* < 0.05, ** *p* < 0.01, *** *p* < 0.001 (Control vs. LN). ^#^ *p* < 0.05, ^##^ *p* < 0.01, ^###^ *p* < 0.001 (LN + NC vs. LN + si-CDO1).

Then, to examine the role of CDO1 in LN, we exposed HK-2 cells to plasma from LN patients at different time points. Western blot analysis revealed a gradual increase in the expression of CDO1 protein, which peaked at 48 h (Figure 3A). Subsequently, CDO1 expression in HK-2 cells was silenced and transfected with siRNA (Figure 3B). Figure 3C,D show that the injury of HK-2 cell was significantly enhanced after LN plasma stimulation, which was markedly alleviated by CDO1 knockdown. Additionally, the LN plasma-induced elevation in apoptosis and EMT was mitigated by downregulating of CDO1 (Figure 3E–J). Figure 3K–N demonstrate that, HK-2 cells treated with LN plasma exhibited an increased level of lipid deposition. However, knockdown of CDO1 significantly reduced cellular lipid deposition.

**Figure 4:**
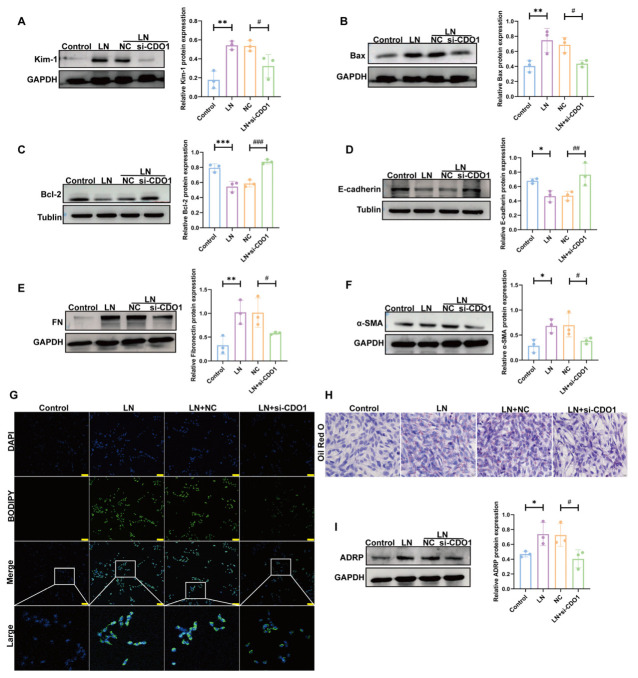
**CDO1 Knockdown alleviated TCMK-1 cell injury induced by LN plasma.** (**A**) Knockdown of CDO1 reduced Kim-1 protein expression (*n* = 3). (**B**,**C**) Apoptosis levels decreased after CDO1 knockdown (*n* = 3). (**D**–**F**) EMT levels were reduced after CDO1 knockdown (*n* = 3). (**G**) BODIPY staining showed alleviated lipid deposition in cells after CDO1 knockdown. The white box indicates the magnified area. The green dots represent lipid droplets. The nucleus is blue. Scale bar: 50 μm. (**H**) Oil red O staining demonstrated reduced lipid deposition in cells after CDO1 knockdown. The red dots represent lipid droplets. The nucleus is blue. Scale bar: 20 μm. (**I**) Knockdown of CDO1 reduced ADRP protein expression (*n* = 3). Data are presented as mean ± SD. * *p* < 0.05, ** *p* < 0.01, *** *p* < 0.001 (Control vs. LN). ^#^ *p* < 0.05, ^##^ *p* < 0.01, ^###^ *p* < 0.001 (LN + NC vs. LN + si-CDO1).

In addition, we stimulated TCMK-1 cells using plasma from LN patients. As shown in Figure 4A, LN plasma stimulation also exacerbated the injury of TCMK-1 cells, while knockdown of CDO1 could significantly alleviate this injury. Furthermore, knockdown of CDO1 also alleviated LN plasma-induced apoptosis, EMT, and lipid deposition (Figure 4B–I).

### 3.3. Knockdown of CDO1 Promoted the Process of Lipid Metabolism and Upregulated the Expression of ACSM3 in LN

**Figure 5:**
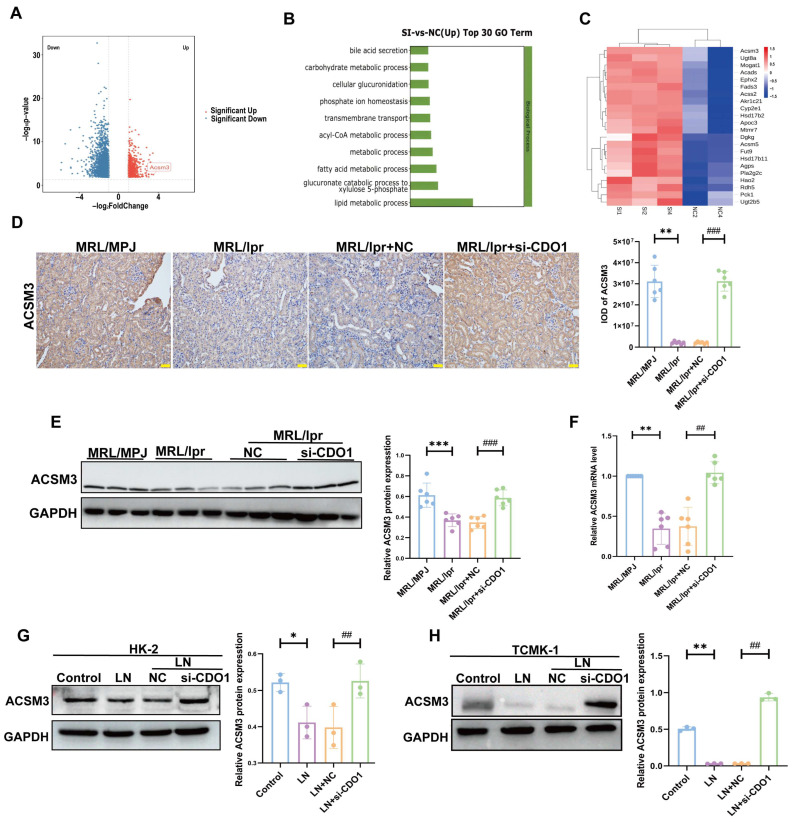
**Knockdown of CDO1 promoted the process of lipid metabolism and upregulated the expression of ACSM3 in LN.** (**A**) Differentially expressed genes in renal tissues between MRL/lpr + NC and MRL/lpr + si-CDO1 mice groups. (**B**) Elevated differentially expressed genes were enriched in different biological processes. (**C**) Differential genes enriched in lipid metabolism. (**D**) IHC showed the expression and localization of ACSM3 in MRL/lpr mice (*n* = 6) (brown areas indicate positivity, nucleus is blue). Scale bar: 50 μm. (**E**,**F**) CDO1 knockdown increased mRNA levels (*n* = 6) and protein expression (*n* = 6) of ACSM3 in MRL/lpr mice. (**G**,**H**) Under the stimulation of 10% LN plasma, knockdown of CDO1 increased ACSM3 protein expression (*n* = 3). Data are presented as mean ± SD. ** *p* < 0.01, *** *p* < 0.001 (MRL/MPJ vs. MRL/lpr). ^##^ *p* < 0.01, ^###^ *p* < 0.001 (MRL/lpr + NC vs. MRL/lpr + si-CDO1). * *p* < 0.05, ** *p* < 0.01, (Control vs. LN). ^##^ *p* < 0.01 (LN + NC vs. LN + si-CDO1).

To further explore the mechanism by which CDO1 mediated renal tubular injury in LN, we performed transcriptomic sequencing of renal cortical tissue from MRL/lpr + NC and MRL/lpr + si-CDO1 mice. As shown in Figure 5A, the analysis of DEGs revealed that ACSM3 was remarkably increased among all the upregulated genes in MRL/lpr + si-CDO1 group compared to MRL/lpr + NC group. GO enrichment analysis indicated that the upregulated DEGs were primarily involved in biological processes related to the lipid and fatty acid metabolism (Figure 5B). In addition, clustering analysis of genes enriched in lipid metabolic process highlighted ACSM3 as the significantly altered gene (Figure 5C). As shown in Figure 5D–H, the mRNA and protein expression of ACSM3 was all decreased in LN group, while upregulated in si-CDO1 group, both in vivo and vitro.

### 3.4. Specific Overexpression of ACSM3 Expression in Renal Tubular Epithelial Cells Alleviated Renal Tubular Injury In Vivo and In Vitro

**Figure 6:**
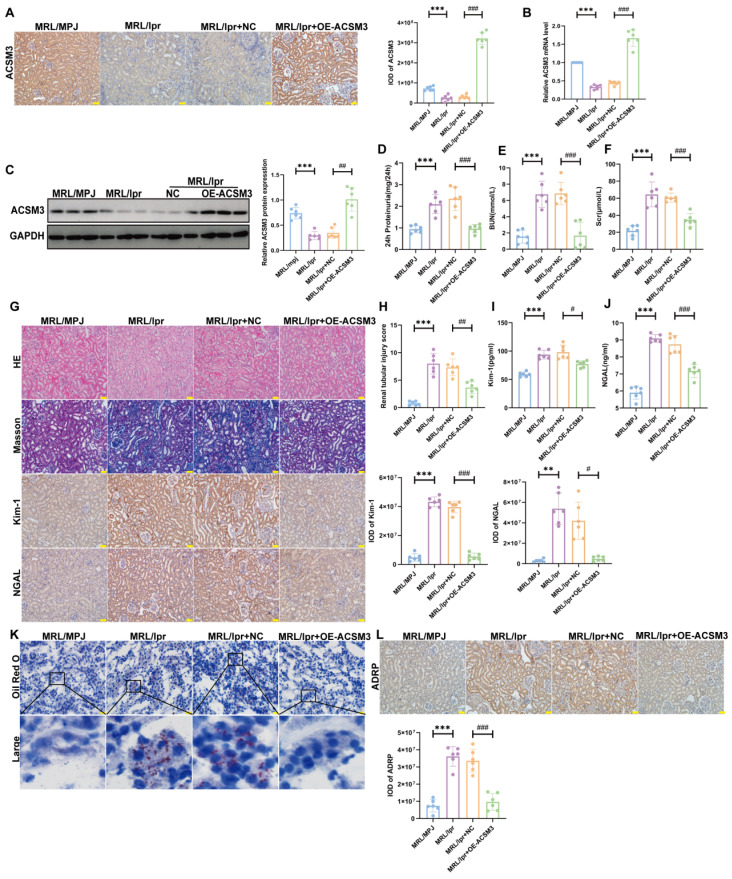
**Specific overexpression of ACSM3 expression in renal tubular alleviated renal tubular injury in MRL/lpr mice.** (**A**) IHC showed the expression and localization of ACSM3 in MRL/lpr mice (*n* = 6) (brown areas indicate positivity, nucleus is blue). Scale bar: 50 μm. (**B**,**C**) ACSM3 overexpression increase mRNA levels (*n* = 6) and protein expression (*n* = 6) of ACSM3 in MRL/lpr mice. (**D**–**F**) ACSM3 overexpression decreased 24 h proteinuria, BUN, and Scr levels in MRL/lpr mice (*n* = 6). (**G**) IHC showed the expression of Kim-1 and NGAL in mouse renal tissue (brown areas indicate positivity). HE staining revealed renal tissue injury in mice. Masson staining showed collagen deposition in mouse renal tissue (blue indicates collagen deposition) (*n* = 6). Scale bars: 50 μm. (**H**) Quantitative analysis of renal tubular injury score in MRL/lpr mice after ACSM3 overexpression (*n* = 6). (**I**,**J**) Urinary Kim-1 and NGAL levels in MRL/lpr mice decreased after ACSM3 overexpression (*n* = 6). (**K**) ACSM3 overexpression alleviated lipid deposition in MRL/lpr mice. The black box indicates the magnified area. The red dot represents the lipid droplet. Scale bar: 20 μm. (**L**) IHC staining showed ADRP expression in MRL/lpr mice (*n* = 6) (brown areas indicate positivity, nucleus is blue). Scale bar: 50 μm. Data are presented as mean ± SD. ** *p* < 0.01, *** *p* < 0.001 (MRL/MPJ vs. MRL/lpr). ^#^ *p* < 0.05, ^##^ *p* < 0.01, ^###^ *p* < 0.001 (MRL/lpr + NC vs. MRL/lpr + OE-ACSM3).

To explore the role of ACSM3 in renal tubular injury and lipid deposition, we specifically overexpressed the expression of renal tubular ACSM3 in MRL/lpr mice. Figure 6A–C show that renal cortical levels of ACSM3 mRNA and protein were significantly increased in MRL/lpr mice following specific overexpression of ACSM3 via Ksp-OE-ACSM3-AAV injection. In addition, ACSM3 overexpression led to significantly improved MRL/lpr mice renal function, along with marked attenuation of tubular injury and interstitial fibrosis in mice (Figure 6D–J). In addition, renal tubular lipid deposition was alleviated in ACSM3-overexpressing mice (Figure 6K,L). These results indicate that overexpression of ACSM3 could alleviate renal tubular injury and renal dysfunction in MRL/lpr mice.

**Figure 7:**
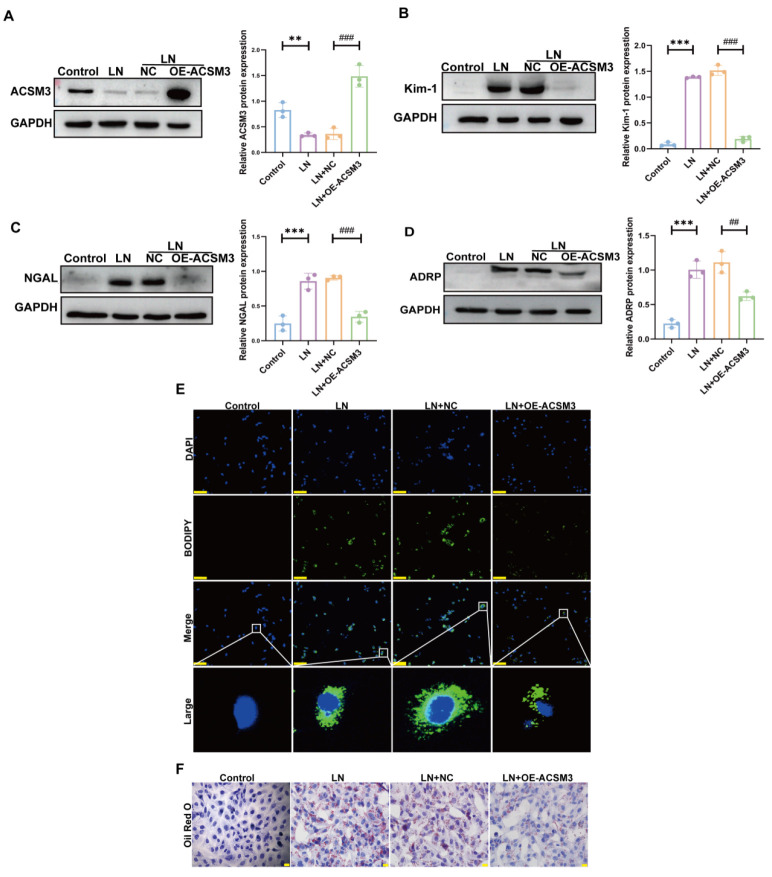
**ACSM3 overexpression alleviated HK-2 cell injury induced by LN plasma.** (**A**) Under the stimulation of 10% LN plasma, the ACSM3 overexpression plasmid increased the expression of ACSM3 in cells. (*n* = 3). (**B**,**C**) Overexpression of ACSM3 reduced Kim-1 and NGAL expression (*n* = 3). (**D**) Overexpression of ACSM3 reduced ADRP expression (*n* = 3). (**E**,**F**) BODIPY staining (The white box indicates the magnified area. The green dots represent lipid droplets. The nucleus is blue) and oil red O (The red dots represent lipid droplets. The nucleus is blue) demonstrated alleviated lipid deposition in cells after ACSM3 overexpression. Scale bars: 20 μm and 50 μm. Data are presented as mean ± SD. ** *p* < 0.01, *** *p* < 0.001 (Control vs. LN). ^##^ *p* < 0.01, ^###^ *p* < 0.001 (LN + NC vs. LN + OE-ACSM3).

**Figure 8:**
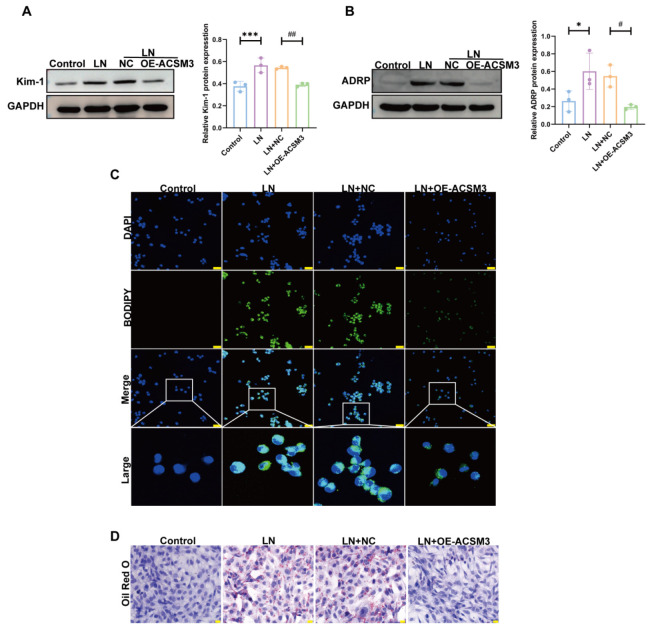
**ACSM3 overexpression alleviated TCMK-1 cell injury induced by LN plasma.** (**A**) Overexpression of ACSM3 reduced Kim-1 expression (*n* = 3). (**B**) Overexpression of ACSM3 reduced ADRP expression (*n* = 3). (**C**,**D**) BODIPY (The white box indicates the magnified area. The green dots represent lipid droplets. The nucleus is blue) and oil red O staining (The red dots represent lipid droplets. The nucleus is blue) demonstrated alleviated lipid deposition in cells after ACSM3 overexpression. Scale bars: 20 μm and 50 μm. Data are presented as mean ± SD. * *p* < 0.05, *** *p* < 0.001 (Control vs. LN). ^#^ *p* < 0.05, ^##^ *p* < 0.01 (LN + NC vs. LN + OE-ACSM3).

In in vitro experiments, we overexpressed ACSM3 in HK-2 cells (Figure 7A). As shown in Figure 7B–F, ACSM3 overexpression significantly alleviated lipid accumulation in HK-2 cells exposed to LN plasma and reduced the expression of ADRP, KIM-1, and NGAL. In parallel, we also examined the effect of ACSM3 overexpression in TCMK-1 cells. The experimental results are consistent with the above results (Figure 8A–D).

### 3.5. The Upregulation of CDO1 Mediated Lipid Deposition of Renal Tubular Cell Partly by Downregulating the Expression of ACSM3 in LN

**Figure 9:**
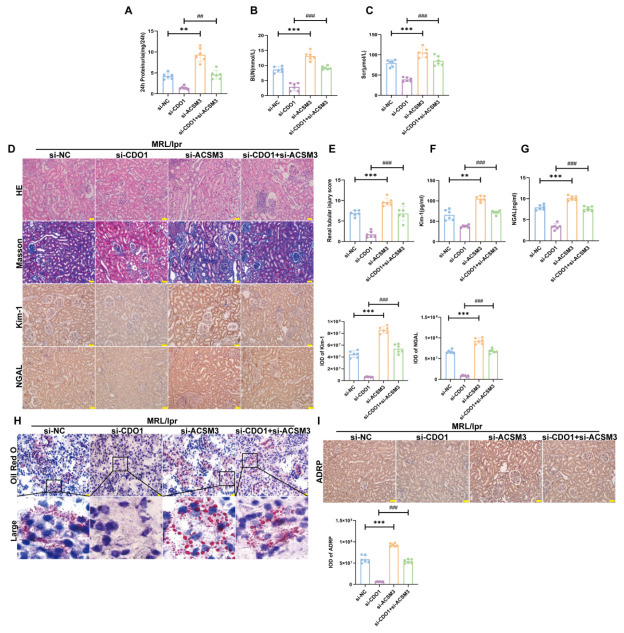
**The upregulation of CDO1 mediated lipid deposition of renal tubular cell partly by downregulating the expression of ACSM3 in MRL/lpr mice.** (**A**–**C**) The 24 h proteinuria, BUN, and scr levels of mice in each group. (*n* = 6). (**D**) IHC showed the expression of Kim-1 and NGAL in mouse renal tissue (brown areas indicate positivity). HE staining revealed renal tissue injury in mice. Masson staining showed collagen deposition in mouse renal tissue (blue indicates collagen deposition) (*n* = 6). Scale bars: 50 μm. (**E**) Quantitative analysis of renal tubular injury scores in each group of mice (*n* = 6). (**F**,**G**) The levels of Kim-1 and NGAL in the urine of mice in each group (*n* = 6). (**H**) Lipid deposition levels in each group of mice. The black box indicates the magnified area. The red dot represents the lipid droplet. Scale bar: 20 μm. (**I**) IHC staining showed ADRP expression in MRL/lpr mice (*n* = 6). (brown areas indicate positivity, nucleus is blue). Scale bar: 50 μm. Data are presented as mean ± SD. ** *p* < 0.01, *** *p* < 0.001 (si-NC vs. si-ACSM3). ^##^ *p* < 0.01, ^###^ *p* < 0.001 (si-CDO1 vs. si-CDO1 + si-ACSM3).

To determine the regulatory effect of CDO1 on ACSM3 in lipid deposition, we specifically performed knockdown of the expressions of CDO1 and ACSM3 in the renal tubules of MRL/lpr mice. Compared with mice in the MRL/lpr+si-CDO1 group, simultaneous knockdown of the expressions of CDO1 and ACSM3 significantly impaired renal function in mice (Figure 9A–C). In addition, simultaneous knockdown of the expressions of CDO1 and ACSM3 aggravated renal tubular injury and renal interstitial fibrosis in mice (Figure 9D–G). It is indicated that knockdown of the expression of ACSM3 in renal tubules could reverse the improvement effect of knockdown CDO1 on renal function and renal tubular injury in MRL/lpr mice. The results of oil red O staining and IHC showed that, simultaneous knockdown of the expressions of CDO1 and ACSM3 could increase lipid deposition in renal tubules and increase the expression of ADRP in renal tubules (Figure 9H,I). It is indicated that knockdown of the expression of ACSM3 in renal tubules could reverse the improvement effect of knockdown CDO1 on lipid deposition in renal tubules of MRL/lpr mice.

**Figure 10:**
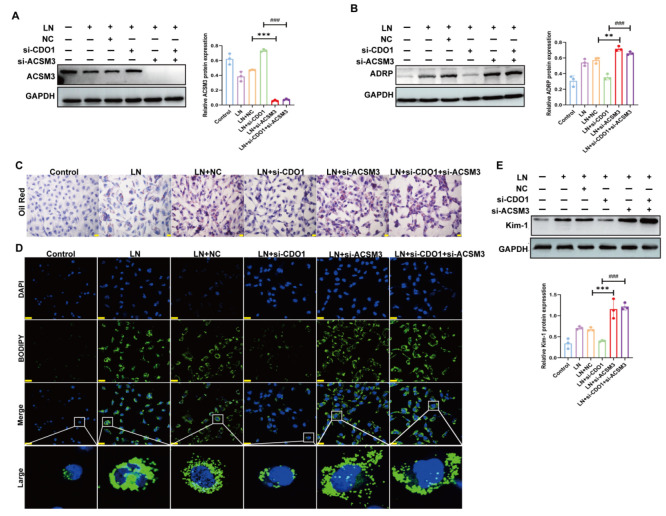
**The upregulation of CDO1 mediated lipid deposition of HK-2 cell partly by downregulating the expression of ACSM3 in LN.** (**A**) Under the stimulation of 10% LN plasma, siRNA downregulated the expression of ACSM3 in HK-2 cells (*n* = 3). (**B**) Knockdown of ACSM3 reversed the effect of CDO1 knockdown on reducing ADRP expression (*n* = 3). (**C**,**D**) Oil red O (The red dots represent lipid droplets. The nucleus is blue.) and BODIPY (The white box indicates the magnified area. The green dots represent lipid droplets. The nucleus is blue.) staining revealed that ACSM3 knockdown reversed the alleviation of lipid deposition in HK-2 cells induced by CDO1 knockdown. (**E**) Knockdown of ACSM3 reversed the effect of CDO1 knockdown on reducing Kim-1 expression (*n* = 3). Scale bars: 20 μm and 25 μm. Data are presented as mean ± SD. ** *p* < 0.01, *** *p* < 0.001 (LN + NC vs. LN + si-ACSM3). ^###^ *p* < 0.001 (LN + si-CDO1 vs. LN + si-CDO1 + si-ACSM3).

Subsequently, we stimulated HK-2 cells with LN plasma while simultaneously knocking down both CDO1 and ACSM3 expression (Figure 10A). Consistent with the in vivo findings, ACSM3 knockdown reversed the alleviative effect of CDO1 knockdown on lipid accumulation in HK-2 cells (Figure 10B–D). Moreover, ACSM3 knockdown also reversed the protective effect of CDO1 knockdown against HK-2 cell injury (Figure 10E).

### 3.6. ACSM3 Contributed to the Lipid Deposition of Renal Tubular Cell by Regulating the Morphology and Function of Mitochondrial

**Figure 11:**
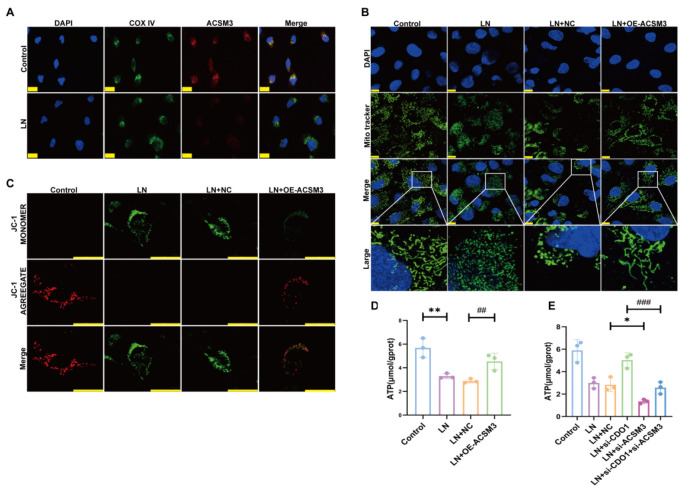
**ACSM3 was contributed to the lipid deposition of renal tubular cell by regulating the morphology and function of mitochondrial.** (**A**) CDO1 and COX IV co-localization in the control and LN groups. Scale bar: 75 μm. (**B**) Mitochondrial morphology improved after ACSM3 overexpression. Scale bar: 10 μm. (**C**) Mitochondrial membrane potential improved after ACSM3 overexpression. Scale bar: 25 μm. (**D**) Under the stimulation of 10% LN plasma, ATP levels increased after ACSM3 overexpression (*n* = 3). (**E**) Knockdown of ACSM3 reversed the effect of CDO1 knockdown on increased ATP levels (*n* = 3). Data are presented as mean ± SD. ** *p* < 0.01 (Control vs. LN). ^##^ *p* < 0.01 (LN+NC vs. LN + OE-ACSM3). * *p* < 0.05 (LN + NC vs. LN + si-ACSM3). ^###^ *p* < 0.001 (LN + si-CDO1 vs. LN + si-CDO1 + si-ACSM3).

Mitochondrial dysfunction is closely associated with lipid accumulation and metabolic imbalance, primarily due to reduced fatty acid oxidation capacity, which in turn exacerbates renal injury and disease progression [16]. To investigate whether ACSM3 regulates abnormal lipid metabolism by influencing mitochondrial morphology and function, we first examined the intracellular localization of ACSM3 using immunofluorescence staining. As shown in Figure 11A, ACSM3 protein was predominantly localized to mitochondria. Furthermore, Mito-Tracker staining revealed that mitochondria in HK-2 cells exposed to LN plasma displayed a fragmented morphology, whereas they shifted to an elongated or rod-shaped morphology following ACSM3 overexpression (Figure 11B). Consistent with this, overexpression of ACSM3 also improved mitochondrial membrane potential in HK-2 cells (Figure 11C). Additionally, ATP production was significantly reduced in HK-2 cells stimulated with LN plasma, an effect that was reversed by ACSM3 overexpression (Figure 11D). More importantly, ATP generation was lower in the LN+si-CDO1+si-ACSM3 group compared with the LN+si-CDO1 group, indicating that downregulation of ACSM3 abolishes the protective effect of CDO1 knockdown on mitochondrial function in renal tubular epithelial cells (Figure 11E).

## 4. Discussion

The role of tubular injury in the progression of LN has become increasingly clear recently. Clinical observations have shown that renal tubular injury contributes to proteinuria, electrolyte imbalances, and renal insufficiency and is closely associated with worsening renal function and poor prognosis in patients with LN [17,18]. However, due to the lack of targeted therapies specifically addressing renal tubular injury, some patients continue to exhibit tubular dysfunction even when glomerular function improves [19]. Moreover, renal tubular injury in LN involves multiple pathological mechanisms [20,21]. Therefore, identifying therapeutic targets to preserve or restore renal tubular function based on these insights has become an urgent clinical need.

To further investigate the mechanism of renal tubular cell in LN, we found CDO1, which upregulated in the proximal tubule and positively correlated with the injury of renal tubule. Recently, CDO1 mediates cell injury by participating in multiple biological processes, in which taurine synthesis and oxidative stress were the important mechanisms [6,22,23]. Zhang, J. et al. found that TRIM47 could interact with CDO1 through its B30.2 domain and promote K48-linked ubiquitination and then lead to reduced CDO1 protein expression, thereby inhibited liver cancer cell ferroptosis [24]. In addition, Yang et al. discovered that a reduction in UHRF1 expression could upregulate the expression of CDO1 through epigenetic mechanisms, thereby increase the susceptibility of hepatocellular carcinoma and cervical cancer cells to ferroptosis [25]. On the other hand, overexpression of CDO1 suppressed the PI3K/AKT signaling pathway and activated the p53 pathway and then promoted apoptosis in breast cancer cells. The above results indicate that high expression of CDO1 can lead to cell injury. In this study, we demonstrated that CDO1 expression was significantly upregulated in the renal tubules of LN, not only in vivo but also in vitro. Moreover, downregulation of CDO1 expression alleviated cellular apoptosis, EMT, and lipid deposition, which indicates that elevated CDO1 expression really plays an important role in renal tubular injury of LN. However, the precise mechanism still unknown.

In recent years, CDO1 has been implicated in the regulation of various metabolic disorders, including insulin resistance, obesity, and neurodegenerative diseases [26,27,28]. For instance, in non-alcoholic fatty liver disease, exercise was shown to induce hepatic CDO1 expression via the cAMP/PKA/CREB signaling pathway [9]. The induced CDO1 promoted the interaction between Camkk2 and AMPK, leading to activation of the AMPK signaling pathway, thereby enhancing the ameliorative effects of exercise on high-fat diet-induced non-alcoholic fatty liver disease [9]. Another study demonstrated that in adipose tissue of obese mice, overexpression of CDO1 promoted energy expenditure, reduced body weight and lipid droplet size, and improved insulin sensitivity, ultimately inhibiting obesity [26]. Additionally, Latorre J found that CDO1 mRNA levels were positively correlated with the expression of genes involved in adipogenesis. Furthermore, in both human preadipocytes and ASC52telo cells, knockdown of the CDO1 gene resulted in decreased expression of adipogenic markers (ADIPOQ, FABP4, FASN, SLC2A4, CEBPA) during adipocyte differentiation [27]. The transcriptomic data from the present study revealed that downregulation of CDO1 expression significantly upregulated genes involved in lipid metabolism, suggesting that elevated CDO1 expression may also contribute to lipid dysregulation during the progression of LN. We speculate that this phenomenon may be related to cell type specificity, the role of the inflammatory environment, and differences in disease stage.

In the following experiment, we surprisingly found the differential expression of ACSM3 in the si-CDO1 group compared to the LN group. ACSM3 belongs to the acyl-CoA synthetase medium-chain family (ACSMs) and is primarily located in the mitochondrial matrix, whose main function is to catalyze the activation of medium-chain fatty acids (MCFAs) through coenzyme A, producing acylated coenzyme A, thereby promoting the synthesis and catabolism of MCFAs [29]. Recently, ACSM3 has been confirmed to be involved in regulating multiple disease processes, including the suppression of tumor development, the induction of adipocyte thermogenesis, and apoptosis [30,31,32]. Studies have shown that inhibiting the expression of ACSM3 accelerated the aging of renal tubular epithelial cells and promoted renal fibrosis [33]. Similarly, our research has confirmed that, in LN, the expression of ACSM3 was decreased, while downregulation of CDO1 expression could increase ACSM3 expression. Furthermore, overexpression of ACSM3 could alleviate lipid deposition and reduce renal tubular epithelial cell injury. However, the rescue experiments demonstrated that downregulation of ACSM3 could reverse the effect of CDO1 knockdown on improving lipid deposition and alleviating cell injury. In conclusion, CDO1 was found to affect lipid metabolic processes by inhibiting the expression of ACSM3. However, how to mediate the lipid deposition needs to be deeply investigated.

The kidney, particularly the proximal renal tubules, is a highly energy-demanding organ that relies on mitochondrial fatty acid oxidation to generate ATP and support tubular reabsorption function. Mitochondrial dysfunction can lead to disrupted lipid metabolism, thereby contributing to the progression of chronic kidney disease (CKD). Previous studies have indicated that downregulation of ACSM3 is closely associated with mitochondrial dysfunction [34]. Xiao et al. reported that ACSM3 expression was reduced in the livers of mice with metabolic syndrome, and this reduction promoted mitochondrial dysfunction and lipid metabolism disorders through the lauric acid–HNF4A–p38 MAPK axis [34]. In line with these findings, our study demonstrated that overexpression of ACSM3 in the context of LN improved mitochondrial structure and function, whereas knockdown of ACSM3 reversed the beneficial effects of CDO1 silencing on mitochondrial morphology and dysfunction. These results suggest that CDO1 exacerbates mitochondrial dysfunction by inhibiting ACSM3 expression, ultimately leading to lipid deposition and renal tubular injury.

Although this study provides new insights into the role of the CDO1–ACSM3 axis in renal tubular injury in lupus nephritis, several limitations remain to be addressed in future experiments. First, the precise molecular mechanism by which CDO1 regulates ACSM3 has not been fully elucidated. We speculate that CDO1 may act at the transcriptional level, potentially by interacting with transcription factors such as PPARγ to suppress ACSM3 transcriptional activity. Second, changes in CDO1 expression could influence DNA methylation status, leading to modifications in the ACSM3 promoter region. Furthermore, CDO1-mediated cysteine metabolism may alter intracellular redox homeostasis, thereby indirectly inhibiting ACSM3 expression through signaling pathways. Finally, within the specific inflammatory microenvironment of lupus nephritis, CDO1 may achieve specific regulation of ACSM3 by modulating key inflammatory signaling pathways such as PI3K/AKT/NF-κB. Meanwhile, the mechanisms underlying the context-dependent roles of CDO1 in different diseases warrant further investigation.

In addition, regarding the potential for clinical translation, this study remains at a preliminary, exploratory stage. First, the expression levels of CDO1 and ACSM3 in easily accessible body fluids such as plasma and urine have not yet been assessed. Their feasibility as non-invasive biomarkers requires subsequent validation using urinary exosomes or plasma samples from LN patients, followed by correlation analysis with traditional indicators such as proteinuria and eGFR. Second, kidney-targeted small-molecule inhibitors of CDO1 or agonists of ACSM3 have not yet been developed, nor has the long-term efficacy and safety of such small-molecule drugs been evaluated in chronic LN models. Meanwhile, targeting the downstream fatty acid oxidation pathway may serve as a novel strategy for addressing metabolic disturbances in LN. Third, the independent prognostic value of CDO1/ACSM3 remains to be established. Future prospective cohort studies are needed to assess their prognostic significance using multivariate regression analysis and to integrate them with conventional indicators to construct a multiparametric prognostic model.

In general, many studies have shown that dysregulation of lipid metabolism plays a key role in the development of LN [20,35]. Our findings indicate that, in LN, CDO1 could cause mitochondrial dysfunction by suppressing the expression of ACSM3, thereby mediating the lipid deposition and injury in renal tubules, which indicates that CDO1 would be expected to be a potential therapeutic target for improving LN.

## 5. Conclusions

Our findings indicate that, in LN, CDO1 causes mitochondrial dysfunction by suppressing the expression of ACSM3, thereby mediating lipid deposition and injury in renal tubules.

## Data Availability

The data that support the findings of this study are available from the corresponding author upon reasonable request.

## Supplementary Materials

The following supporting information can be downloaded at: https://www.mdpi.com/article/10.3390/cells15050461/s1, Table S1: Mouse Group and Treatments.

## References

[1] Hong S., Healy H., Kassianos A.J. (2020). The emerging role of renal tubular epithelial cells in the immunological pathophysiology of lupus nephritis. Front. Immunol..

[2] Moroni G., Porata G., Raffiotta F., Quaglini S., Frontini G., Sacchi L., Binda V., Calatroni M., Reggiani F., Banfi G. (2021). Beyond ISN/RPS lupus nephritis classification: Adding chronicity index to clinical variables predicts kidney survival. Kidney360.

[3] Fu D., Senouthai S., Wang J., You Y. (2019). FKN facilitates HK-2 cell EMT and tubulointerstitial lesions via the wnt/β-catenin pathway in a murine model of lupus nephritis. Front. Immunol..

[4] Cantaluppi V., Quercia A.D., Dellepiane S., Ferrario S., Camussi G., Biancone L. (2014). Interaction between systemic inflammation and renal tubular epithelial cells. Nephrol. Dial. Transplant..

[5] Hirschberger L.L., Daval S., Stover P.J., Stipanuk M.H. (2001). Murine cysteine dioxygenase gene: Structural organization, tissue-specific expression and promoter identification. Gene.

[6] Chen M., Zhu J.Y., Mu W.J., Guo L. (2023). Cysteine dioxygenase type 1 (CDO1): Its functional role in physiological and pathophysiological processes. Genes Dis..

[7] Yang J., Sun L., Liu X., Huang C., Peng J., Zeng X., Zheng H., Cen W., Xu Y., Zhu W. (2023). Targeted demethylation of the CDO1 promoter based on CRISPR system inhibits the malignant potential of breast cancer cells. Clin. Transl. Med..

[8] Hao S., Yu J., He W., Huang Q., Zhao Y., Liang B., Zhang S., Wen Z., Dong S., Rao J. (2017). Cysteine dioxygenase 1 mediates erastin-induced ferroptosis in human gastric cancer cells. Neoplasia.

[9] Chen M., Zhu J.Y., Mu W.J., Luo H.Y., Li Y., Li S., Yan L.J., Li R.Y., Guo L. (2023). Cdo1-Camkk2-AMPK axis confers the protective effects of exercise against NAFLD in mice. Nat. Commun..

[10] Shi K., Jia B., Li Y., Feng X., Sun X., Liu Q., Zhang W., Tian Y., Miao X., Liu Y. (2025). Loss of TRIM44 promotes renal cell carcinoma progression by regulating K48-linked ubiquitination of vimentin. J. Biol. Chem..

[11] Tian Y., Guo H., Miao X., Xu J., Yang R., Zhao L., Liu J., Yang L., Gao F., Zhang W. (2020). Nestin protects podocyte from injury in lupus nephritis by mitophagy and oxidative stress. Cell Death Dis..

[12] Miao X., Tian Y., Wu L., Zhao H., Liu J., Gao F., Zhang W., Liu Q., Guo H., Yang L. (2022). CircRTN4 aggravates mesangial cell dysfunction by activating the miR-513a-5p/FN axis in lupus nephritis. Lab Investig..

[13] Linkermann A., Skouta R., Himmerkus N., Mulay S.R., Dewitz C., De Zen F., Prokai A., Zuchtriegel G., Krombach F., Welz P.-S. (2014). Synchronized renal tubular cell death involves ferroptosis. Proc. Natl. Acad. Sci. USA.

[14] Liu J., Zhao T., Cui H., Tian Y., Miao X., Xing L., Wang X., Huang J., Liu Q., Zhang W. (2025). HMGB1 encapsulated in podocyte-derived exosomes plays a central role in glomerular endothelial cell injury in lupus nephritis by regulating TRIM27 expression. Lab Investig..

[15] Gu C., Gao F., Zhang S., Kang L., Zhang W., Feng X., Liu J., Tian Y., Wei Q., Du Y. (2023). Role of SUMOylation of STAT1 in tubular epithelial-mesenchymal transition induced by high glucose. Mol. Med. Rep..

[16] Ge M., Fontanesi F., Merscher S., Fornoni A. (2020). The vicious cycle of renal lipotoxicity and mitochondrial dysfunction. Front. Physiol..

[17] Lan-Ting H., You-Ming C., Li-Xin W., Chen W., Xiao-Yan Z., Hong-Yan H. (2020). Clinicopathological factors for tubulointerstitial injury in lupus nephritis. Clin. Rheumatol..

[18] Hui W.F., Chan V.P.Y., Cheung W.L., Ku S.W., Hon K.L. (2024). The impact of tubular dysfunction and its relationship with acute kidney injury in children. Pediatr. Nephrol..

[19] Malvar A., Pirruccio P., Alberton V., Lococo B., Recalde C., Fazini B., Nagaraja H., Indrakanti D., Rovin B.H. (2017). Histologic versus clinical remission in proliferative lupus nephritis. Nephrol. Dial. Transplant..

[20] Xue L., Zhang Y., Xu J., Lu W., Wang Q., Fu J., Liu Z. (2021). Anti-tweak antibody alleviates renal interstitial fibrosis by increasing pgc-1α expression in lupus nephritis. J. Inflamm. Res..

[21] Mao Z., Tan Y., Tao J., Li L., Yu F., Zhao M. (2022). mTORC1 activation induced proximal tubular damage via the pentose phosphate pathway in lupus nephritis. Free Radic. Biol. Med..

[22] Ueki I., Roman H.B., Valli A., Fieselmann K., Lam J., Peters R., Hirschberger L.L., Stipanuk M.H. (2011). Knockout of the murine cysteine dioxygenase gene results in severe impairment in ability to synthesize taurine and an increased catabolism of cysteine to hydrogen sulfide. Am. J. Physiol. Endocrinol. Metab..

[23] Ma G., Zhao Z., Qu Y., Cai F., Liu S., Liang H., Zhang R., Deng J. (2022). Cysteine dioxygenase 1 attenuates the proliferation via inducing oxidative stress and integrated stress response in gastric cancer cells. Cell Death Discov..

[24] Zhang J., Yimamu M., Cheng Z., Ji J., Wu L., Feng J., Xu X., Wu J., Guo C. (2024). TRIM47-CDO1 axis dictates hepatocellular carcinoma progression by modulating ferroptotic cell death through the ubiquitin‒proteasome system. Free Radic. Biol. Med..

[25] Yang R., Zhou Y., Zhang T., Wang S., Wang J., Cheng Y., Li H., Jiang W., Yang Z., Zhang X. (2023). The transcription factor HBP1 promotes ferroptosis in tumor cells by regulating the UHRF1-CDO1 axis. PLoS Biol..

[26] Guo Y.Y., Li B.Y., Xiao G., Liu Y., Guo L., Tang Q.Q. (2022). Cdo1 promotes PPARγ-mediated adipose tissue lipolysis in male mice. Nat. Metab..

[27] Latorre J., Mayneris-Perxachs J., Oliveras-Cañellas N., Ortega F., Comas F., Fernández-Real J.M., Moreno-Navarrete J.M. (2022). Adipose tissue cysteine dioxygenase type 1 is associated with an anti-inflammatory profile, impacting on systemic metabolic traits. EBioMedicine.

[28] Perry T.L., Norman M.G., Yong V.W., Whiting S., Crichton J.U., Hansen S., Kish S.J. (1985). Hallervorden-Spatz disease: Cysteine accumulation and cysteine dioxygenase deficiency in the globus pallidus. Ann. Neurol..

[29] Watkins P.A., Maiguel D., Jia Z., Pevsner J. (2007). Evidence for 26 distinct acyl-coenzyme A synthetase genes in the human genome. J. Lipid Res..

[30] Yang X., Wu G., Zhang Q., Chen X., Li J., Han Q., Yang L., Wang C., Huang M., Li Y. (2022). ACSM3 suppresses the pathogenesis of high-grade serous ovarian carcinoma via promoting AMPK activity. Cell Oncol..

[31] Wang J., Sun Y., Wu R. (2025). ACSM3 suppresses ovarian cancer progression by inactivating the IFN-γ/JAK/STAT3 signaling pathway. Adv. Biol..

[32] Cheng L., Shi L., He C., Wang C., Lv Y., Li H., An Y., Dai H., Duan Y., Zhang H. (2022). Rutin-activated adipose tissue thermogenesis is correlated with increased intestinal short-chain fatty acid levels. Phytother. Res..

[33] Li L., Wu Y., Ma L., Fu P. (2023). #3467 inhibition of acyl-coa synthetase medium chain family member 3 promotes renal fibrosis by accelerating cellular senescence in tubular epithelial cells. Nephrol. Dial. Transplant..

[34] Xiao X., Li R., Cui B., Lv C., Zhang Y., Zheng J., Hui R., Wang Y. (2024). Liver ACSM3 deficiency mediates metabolic syndrome via a lauric acid-HNF4α-p38 MAPK axis. EMBO J..

[35] Hu C., Du Y., Xu X., Li H., Duan Q., Xie Z., Wen C., Han X. (2021). Lipidomics revealed aberrant metabolism of lipids including fahfas in renal tissue in the progression of lupus nephritis in a murine model. Metabolites.

