# Supplementary material for: The CDO1–ACSM3 Axis Mediates Renal Tubule Lipid Deposition and Injury by Causing Mitochondrial Dysfunction in Lupus Nephritis
Source: Cells. 2026 Mar 4;15(5):461. doi: 10.3390/cells15050461 (PMC12984251; doi:10.3390/cells15050461)
Supplement: Supplementary file 1 [file cells-15-00461-s001.zip › cells-4119005-supplementary.pdf]

Supplementary table S1

Table S1 Mouse Group and Treatments

Figure	Group	Mouse Strain	Treatment	Injected Viral Vector
-	MRL/MPJ	Normal control mice	No intervention	No viral injection
-	MRL/lpr	MRL/lpr Lupus-prone mice	No intervention	No viral injection
Figure 2/Figure 5	MRL/lpr+NC	MRL/lpr Lupus-prone mice	Negative control (non-targeted knockdown)	Ksp-si-NC-AAV (non-specific control vector)
	MRL/lpr+si-CDO1	MRL/lpr Lupus-prone mice	CDO1 knockdown	Ksp-si-CDO1-AAV (viral vector for CDO1 knockdown
Figure 6	MRL/lpr+NC	MRL/lpr Lupus-prone mice	Negative control (non-targeted overexpression)	Ksp-NC-AAV (non-specific control vector)
	MRL/lpr+OE-ACSM3	MRL/lpr Lupus-prone mice	ACSM3 overexpression	Ksp-OE-ACSM3-AAV (viral vector for ACSM3 overexpression)
Figure 9	MRL/lpr+si-NC	MRL/lpr Lupus-prone mice	Negative control (non-targeted knockdown)	Ksp-NC-AAV (non-specific control vector)
	MRL/lpr+si-CDO1	MRL/lpr Lupus-prone mice	CDO1 knockdown	Ksp-si-CDO1-AAV (CDO1 knockdown)
	MRL/lpr+si-ACSM3	MRL/lpr Lupus-prone mice	ACSM3 knockdown	Ksp-si-ACSM3-AAV (viral vector for ACSM3 knockdown)
	MRL/lpr+si-CDO1 +si-ACSM3	MRL/lpr Lupus-prone mice	CDO1 knockdown +ACSM3 knockdown	Ksp-si-CDO1-AAV (CDO1 knockdown) + Ksp-si-ACSM3-AAV (ACSM3 knockdown)
